# Cell-mediated immunity to encephalitogenic factor (MMI test) in women with cervical dysplasia and carcinoma in situ: the effects of serum.

**DOI:** 10.1038/bjc.1978.220

**Published:** 1978-09

**Authors:** D. J. Flavell, A. Singer, C. W. Potter

## Abstract

Lymphocyte sensitivity to encephalitogenic factor (EF) was measured with the macrophage migration inhibition (MMI) test in 60 women with dysplasia or carcinoma in situ of the cervix, in 10 women with invasive cervical carcinoma, and in 20 women with a variety of benign gynaecological conditions. Significant migration inhibition with EF (P less than 0.01) was seen with lymphocytes taken from 7/13 (54%) women with mild and/or moderate dysplasia, from 22/47 (47%) women with severe dysplasia and/or carcinoma in situ, from 6/10 (60%) women with invasive cervical carcinoma and from 3/20 (15%) women with benign gynaecological conditions. Autologous serum was seen to abrogate EF-mediated migration inhibition in 3/4 sensitized women with mild and/or moderate dysplasia, in 5/7 sensitized women with severe dysplasia and/or csrcinoma in situ and in 2/3 sensitized women with invasive cervical carcinoma. Autologous serum from 2 sensitized women with benign gynaecological conditions did not abrogate the response of their lymphocytes to EF.


*Br. J. Cancer* (1978) **38**, 396

### CELL-MEDIATED IMMUNITY TO ENCEPHALITOGENIC FACTOR (MMI TEST) IN WOMEN WITH CERVICAL DYSPLASIA AND CARCINOMA *IN SITU*: THE EFFECTS OF SERUM

D. J. FLAVELL*[1], A. SINGER[2] AND C. W. POTTER[3]

*From the [1]Department of Pathology, Western Park Hospital, Sheffield S10 2SJ, the [2]Department of Obstetrics & Gynaecology, University of Sheffield and the Jessop Hospital for Women, and the [3]Department of Virology, University of Sheffield Medical School, Sheffield S10 2RX*

Received 19 May 1978 Accepted 14 June 1978

**Summary.—Lymphocyte sensitivity to encephalitogenic factor (EF) was measured with the macrophage migration inhibition (MMI) test in 60 women with dysplasia or carcinoma *in situ* of the cervix, in 10 women with invasive cervical carcinoma, and in 20 women with a variety of benign gynaecological conditions. Significant migration inhibition with EF ($P<0{\cdot}01$) was seen with lymphocytes taken from 7/13 (54%) women with mild and/or moderate dysplasia, from 22/47 (47%) women with severe dysplasia and/or carcinoma *in situ*, from 6/10 (60%) women with invasive cervical carcinoma and from 3/20 (15%) women with benign gynaecological conditions.**

**Autologous serum was seen to abrogate EF-mediated migration inhibition in 3/4 sensitized women with mild and/or moderate dysplasia, in 5/7 sensitized women with severe dysplasia and/or carcinoma *in situ* and in 2/3 sensitized women with invasive cervical carcinoma. Autologous serum from 2 sensitized women with benign gynaecological conditions did not abrogate the response of their lymphocytes to EF.**

SINCE the original observation by Field & Caspary (1970) that lymphocytes respond to encephalitogenic factor (EF) in the macrophage electrophoretic mobility (MEM) test, there is now evidence to suggest that this response is directed against a common neoantigen or common neoantigens on the tumour cell surface (Caspary & Field, 1971; Dickinson *et al.*, 1973; Coates & Carnegie, 1975). Similar results have been reported using the macrophage migration inhibition (MMI) test (Light *et al.*, 1975) but this test may be less sensitive than the MEM test (Hughes & Paty, 1971). Moreover, there is further evidence that the lymphocyte response to EF may occur many years before the clinical appearance of tumour (Field *et al.*, 1972; Pritchard *et al.*, 1976). Thus Singer and co-workers (1975) and Porzsolt *et al.* (1975) have shown that a large proportion of women with premalignant cervical lesions and carcinoma *in situ* show a lymphocyte response to EF. Furthermore Flavell *et al.* (1978), using an animal model, have demonstrated that about half the rats with carcinogen-induced dysplastic hepatic lesions show a spleen-cell response to EF in the MMI test. In view of this evidence, it seems possible that the demonstration of a cell-mediated immune response to EF may prove useful in detecting not only cervical premalignant lesions but premalignant lesions at other sites.

In the present paper we report on the incidence of a cell-mediated immune response to EF as determined with the MMI test in women with premalignant and malignant cervical lesions, and in a group of women with a variety of benign gynaecological conditions. In addition, the effects of autologous serum on the lympho-

* Current address: Department of Radiology, Siriraj Hospital, Mahidol University, Bangkok 7, Thailand.

cyte response to EF was investigated in some of the patients.

### MATERIALS AND METHODS

*Patients.*—A total of 60 women with either dysplasia or carcinoma *in situ* of the cervix were selected for study. Diagnosis was made histologically from punch-biopsy specimens taken at colposcopy, according to the criteria described by the R.C.O.G. panel (Govan *et al.*, 1969) and by W.H.O. (Riotton and Christopherson, 1973). Because of the difficulties often encountered in discriminating histologically between mild and moderate dysplasia or severe dysplasia and carcinoma *in situ* (Koss, 1978) these 2 sets of lesions were grouped together in the study. When both types of lesion were present, the more severe was accepted as representative. The group of 13 women with mild and/or moderate dysplasia had a mean age of 24 years (range 19–42) whilst the group of 47 women with severe dysplasia and/or carcinoma *in situ* had a mean age of 33 years (range 18–56). A group of 10 women with invasive cervical carcinoma were also included in the study; they had a mean age of 46 years (range 30–65). A group of 20 women with various benign gynaecological conditions was also included in the study. Their individual diagnoses are given in Table I. They had a mean age of 32 years (range 16–59).

*MMI test.*—Lymphocytes harvested from peripheral blood (Harris and Ukaejiofo, 1970) were tested for response to EF using the direct MMI test. Full details of this test system have been given elsewhere (Flavell and Potter, 1978). Briefly, lymphocytes and peritoneal-exudate cells from guinea-pigs stimulated with i.p. liquid paraffin (Rees and Potter, 1973) were mixed in a ratio of 1:5 and packed into 10$\mu$l capillary tubes. Cut capillary tubes were incubated in Medium 199 containing 10% heat-inactivated foetal calf serum in the absence or in the presence of 100 $\mu$g of EF for 24 h at 37°C. Duplicate control wells each containing 3 capillary tubes were set up for each treatment. Areas of macrophage migration were estimated at 24 h, and the percentage of migration inhibition with EF calculated. The significance of migration inhibition was assessed with Student's $t$ test. A value of $P<0{\cdot}01$ was considered indicative of significant migration inhibition. The effects of heat-inactivated autologous serum upon EF-mediated migration inhibition were investigated by including serum in duplicate sets of wells at a concentration of 10% with and without EF in the medium.

TABLE I.—*MMI by lymphocytes from women with cervical dysplasia, carcinoma* in situ, *invasive cervical carcinoma, and benign gynaecological conditions, in the presence of EF*

| Diagnosis | Number tested | No. (%) showing significant MMI ($P<0{\cdot}01$) |
|---|---|---|
| Mild and/or moderate dysplasia | 13 | 7 (54) |
| Severe dysplasia and/or Ca *in situ* | 47 | 22 (47) |
| Invasive cervical carcinoma | 10 | 6 (60) |
| Benign gynaecological conditions* | 20 | 3 (15) |

* Patients with the following conditions were used as controls: first trimester pregnancy (6), large cervical ectropion ("erosion") (5), pelvic inflammatory disease (5), prolapse (1), anovulation (1), ovarian cyst (1), and a cervical polyp (1).

### RESULTS

### *MMI with EF*

Table I shows the number of women from each group whose lymphocytes showed significant migration inhibition in the presence of EF. The percentage of migration inhibition with EF seen for each individual is presented as a scatter-graph in the Fig. Of the 13 women classified as mild and/or moderate dysplasia, 7 (54%) showed a response to EF; and of 47 women classified as severe dysplasia and/or carcinoma *in situ*, 22 (47%) showed a lymphocyte response to EF. Lymphocytes from 6/10 (60%) women with invasive cervical carcinoma gave significant migration inhibition with EF. Of the 20 women with benign gynaecological conditions, 3 (15%) showed significant migration inhibition with EF. There was one woman with a cervical erosion, one woman with chronic cervicitis and one woman in the first trimester of pregnancy.

FIG.—Percentage of significant (○) and non-significant (●) macrophage migration inhibition with EF by lymphocytes from women with cervical dysplasia, carcinoma *in situ*, invasive cervical carcinoma and women with benign gynaecological conditions.

*Serum inhibition of MMI*

The effects of autologous serum upon EF-mediated migration inhibition were investigated in 25 of the women with dysplasia and/or carcinoma *in situ*, in 7 women with invasive cervical carcinoma, and 9 women with benign gynaecological conditions. The results are shown in Table II. Of the 4 women investigated in the mild and/or moderate-dysplasia group, 3 showed significant migration inhibition with EF in the absence of serum, whilst in the presence of autologous serum only 1 showed such a response. Of the 21 women investigated in the severe dysplasia and/or carcinoma *in situ* group, 7 showed significant migration inhibition with EF in the absence of serum, whilst in the presence of autologous serum this response was abolished in 5 of them. However, autologous serum from one woman in this group increased the observed migration-inhibition response to EF from a non-significant level in the absence of serum to a significant level in the presence of serum. Of the 7 women investigated with invasive cervical carcinoma, 3 showed significant migration inhibition with EF in the absence of serum, whilst in the presence of serum this response was abolished in 2 of them. Autologous serum from 2 sensitized women with benign gynaecological conditions did not abrogate the response of their lymphocytes to EF. However, it was observed that autologous serum from one woman with cervical squamous metaplasia increased the observed migration inhibition response to EF from a non-significant level in the absence of serum to a significant level in the presence of serum.

TABLE II.—*MMI by lymphocytes from women with cervical dysplasia, carcinoma* in situ, *invasive cervical carcinoma and benign gynaecological conditions, in the presence of EF with and without autologous serum*

| Diagnosis | No. tested | No. (%) showing significant MMI ($P<0{\cdot}01$) Without serum | With serum |
|---|---|---|---|
| Mild and/or moderate dysplasia | 4 | 3 (75) | 1 (25) |
| Severe dysplasia and/or Ca *in situ* | 21 | 7 (33) | 3 (14) |
| Invasive cervical carcinoma | 7 | 3 (43) | 1 (14) |
| Benign gynaecological conditions* | 9 | 2 (22) | 3 (33) |

* Group includes: 4 cases with cervical ectropion (erosions) and 5 with pelvic inflammatory disease.

### COMMENT

The results of the present study clearly demonstrate that a large proportion of women with premalignant cervical lesions show a demonstrable lymphocyte response to EF when tested with the MMI test. Singer and his associates (1975), using the MMI test, also demonstrated that 70% of women with carcinoma *in situ* and 42% of women with dysplasia showed a lymphocyte response to EF, and sensitization to EF has been demonstrated in other malignant diseases, using the same test (Shelton *et al.* 1975; Light *et al.*, 1975). The incidence of lymphocyte response to EF observed in the present study was lower than that reported by Singer and coworkers (1975). This discrepancy may be due to differences in the classification system employed in the 2 studies; in the present study, women with mild and moderate dysplasia and those with severe dysplasia and carcinoma *in situ* were grouped together.

The observed incidence of lymphocyte response to EF in women with premalignant cervical lesions is considerably higher than for that in women with a variety of benign gynaecological conditions, and is about the same as that in women with invasive cervical carcinoma. A recent report by Flavell & Potter (1978) has shown that lymphocytes from 63% of cancer patients studied respond to EF in the MMI test, whilst lymphocytes from 32% of individuals with a variety of non-malignant conditions showed a similar response. Thus, the incidence of lymphocyte response to EF seen in women with premalignant cervical lesions falls about halfway between those seen in cancer patients and patients with benign conditions.

Caspary & Field (1971), using the macrophage electrophoretic mobility (MEM) test, have demonstrated that lymphocytes from cancer patients not only respond to EF, but also to an acid extract of malignant tissues termed "cancer basic protein" (CaBP). Further studies have shown that EF and CaBP have remarkable chemical similarities (Dickinson & Caspary, 1973) and it has been suggested that the EF and CaBP molecules share common antigenic determinants (Coates & Carnegie, 1975; McDermott *et al.*, 1974; Flavell & Potter, 1978). This has led to the proposal that the lymphocyte response to EF in malignant disease might represent a cell-mediated immune response directed against neoantigens on the tumour-cell surface which share an antigenic determinant or determinants with EF. Thus, it is conceivable that the lymphocyte response to EF seen in women with premalignant lesions may be due to the appearance of a neoantigen or neoantigens on the dysplastic epithelial cell surface, which is immunologically cross-reactive with EF; indeed, the appearance of a lymphocyte response to EF in these women may prove to be an indicator of the malignant potential of these lesions.

Serum from most of the women with cervical dysplasia and carcinoma *in situ* who showed a lymphocyte response to EF abolished this response *in vitro*. Flavell & Potter (1978) have recently shown that serum from most cancer patients showing lymphocyte response to EF is capable of abolishing EF-mediated migration inhibition. Similarly, serum flow patients with non-malignant conditions who showed a lymphocyte response to EF, abolished this response in about half of these individuals. However, it is difficult to make a direct comparison between the incidence of serum abrogation of the lymphocyte response to EF seen in the present study and that seen by Flavell & Potter (1978), in view of the small number of women with cervical dysplasia and carcinoma *in situ* investigated in the present study. However, this preliminary investigation suggests that women with premalignant cervical lesions might show a similar incidence of serum abrogation of the lymphocyte response to EF to that seen in cancer patients. Field & Caspary (1972) have shown that a component(s) present in serum from cancer patients is capable of depressing the lymphocyte response to EF and CaBP in the MEM test. Further studies have shown that the $\alpha_2$ macroglobulin component of serum is responsible for this depression (Ford *et al.*, 1973). Furthermore, studies by Bernard & Lamoureux (1975) have shown that remixing $\alpha_2$ macroglobulin with EF abolishes the encephalitogenic and thus antigenic potency of the EF molecule. It is possible that the serum-blocking effects found in the present study may be due to the release of $\alpha_2$-macroglobulin-like components to the circulation, which may perform some immunoregulatory function after tissue damage and subsequent release of normal tissue antigen. Thus, the production of serum factor(s) which abrogate EF sensitivity may have important implications in the immune response to premalignant and malignant lesions, and the detection of these factors may be of practical value in the laboratory diagnosis of such lesions.

We would like to thank the consultant and nursing staff of the Jessop Hospital for Women, Sheffield, for their patience and cooperation during the course of this study. One of us (D.J.F.) was supported by a grant from the Yorkshire branch of the British Cancer Research Campaign. Further assistance was received by a grant from L.R. Industries (London).

### REFERENCES

BERNARD, C. C. & LAMOUREUX, G. (1975) Inhibition of serum of encephalitogenic activity of myelin basic protein: nature of the serum factor responsible. *Cell. Immunol.*, **16**, 182.

CASPARY, E. A. & FIELD, E. J. (1971) Specific lymphocyte sensitisation in cancer: Is there a common antigen in human malignant neoplasia? *Br. Med. J.*, ii, 613.

COATES, A. S. & CARNEGIE, P. R. (1975) Immunological cross reactivity between basic proteins of myelin and cancer. 1. Lymphocyte tranformations studies in immunised guinea pigs. *Clin. Exp. Immunol.*, **22**, 16.

DICKINSON, J. P., CASPARY, E. A. & FIELD, E. J. (1973) A common tumour specific antigen 1. Restriction *in vivo* to malignant neoplastic tissue. *Br. J. Cancer*, **27**, 99.

DICKINSON, J. P. & CASPARY, E. A. (1973) The chemical nature of cancer basic protein. *Br. J. Cancer*, **28** (Suppl. 1), 224.

FIELD, E. J. & CASPARY, E. A. (1970) Lymphocyte sensitisation: an *in vitro* test for cancer? *Lancet*, ii, 1137.

FIELD, E. J., CASPARY, E. A. & SHEPHERD, R. H. T. (1972) Immunodiagnosis of cancer. *Br. Med. J.*, iii, 641.

FIELD, E. J. & CASPARY, E. A. (1972) Lymphocyte sensitisation in advanced malignant disease: a study of serum lymphocyte depressive factor. *Br. J. Cancer*, **26**, 164.

FLAVELL, D. J. & POTTER, C. W. (1978) Cellular immunity to encephalitogenic factor in man, as measured by the macrophage migration inhibition test: the effects of serum. *Br. J. Cancer*, **37**, 15.

FLAVELL, D. J., GOEPEL, J., POTTER, C. W. & CARR, I. (1978) Cellular immunity to encephalitogenic factor as measured with the macrophage migration inhibition test during tumour induction and growth. *Br. J. Cancer*, **37**, 818.

FORD, W. H., CASPARY, E. A. & SHENTON, B. (1973) Purification and properties of a lymphocyte inhibition factor from human serum. *Clin. Exp. Immunol.*, **15**, 169.

GOVAN, A. D. T., HAINES, R. M., LANGLEY, F. A., TAYLOR, C. W. & WOODCOCK, A. S. (1969) The histology and cytology of changes in the epithelium of the cervix uteri. *J. Clin. Pathol.*, **22**, 383.

HARRIS, R. & UKAEJIOFO, E. O. (1970) Tissue typing using a routine one-step lymphocyte separation procedure. *Br. J. Haematol.*, **18**, 229.

HUGHES, D. & PATY, D. W. (1971) Lymphocyte sensitivity in cancer. *Br. Med. J.*, ii, 770.

KOSS, L. G. (1978) Dysplasia; a real concept or misnomer. *Obstet. Gynaecol.*, **51**, 374.

LIGHT, P. A., PREECE, A. W. & WALDRON, H. A. (1975) Studies with macrophage migration inhibition (MMI) test in patients with malignant disease. *Clin. Exp. Immunol.*, **22**, 279.

MCDERMOTT, J. R., CASPARY, E. A. & DICKINSON, J. P. (1974) Antigen cross reactivity in the macrophage electrophoretic mobility test. A study using cellular affinity chromatography. *Clin. Exp. Immunol.*, **17**, 103.

PORZSOLT, F., MULBERGER, C. T. & AX, W. (1975) Electrophoretic mobility test (EMT). II. Is there a correlation between the clinical diagnosis and immunologic test for precancerous diseases? *Behring Inst. Mitt.*, **57**, 137.

PRITCHARD, J. A. V., MOORE, J. L., SUTHERLAND, W. H. & JOSLIN, C. A. F. (1976) Clinical assessment of the MOD-MEM test cancer test in controls with non-malignant diseases. *Br. J. Cancer*, **34**, 1.

REES, R. C. & POTTER, C. W. (1973) Immune response to adenovirus 12-induced antigens as measured *in vitro* by the macrophage migration inhibition test. *Eur. J. Cancer*, **8**, 497.

RIOTTON, G. & CHRISTOPHERSON, W. (1973) *Cytology of the Female Genital Tract*, W.H.O., Geneva.

SHELTON, J. B., POTTER, C. W. & CARR, I. (1975) Cellular immunity to myelin basic protein in man and in animal model systems as measured by the macrophage migration inhibition test. *Br. J. Cancer*, **31**, 528.

SINGER, A., SHELTON, J., HILL, S. & POTTER, C. W. (1975) Cellular immunity to myelin basic protein in women with dysplasia and carcinoma *in situ* of the cervix. *Br. J. Obstet. Gynaecol.*, **82**, 820.


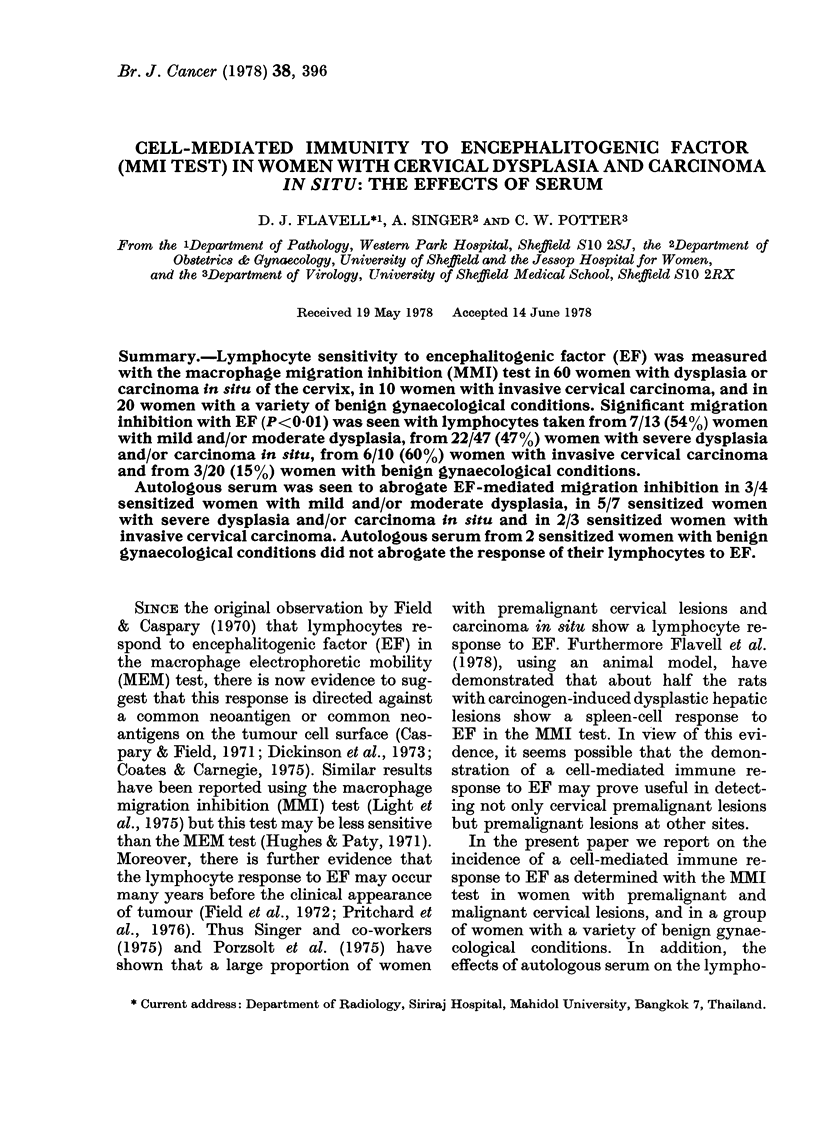

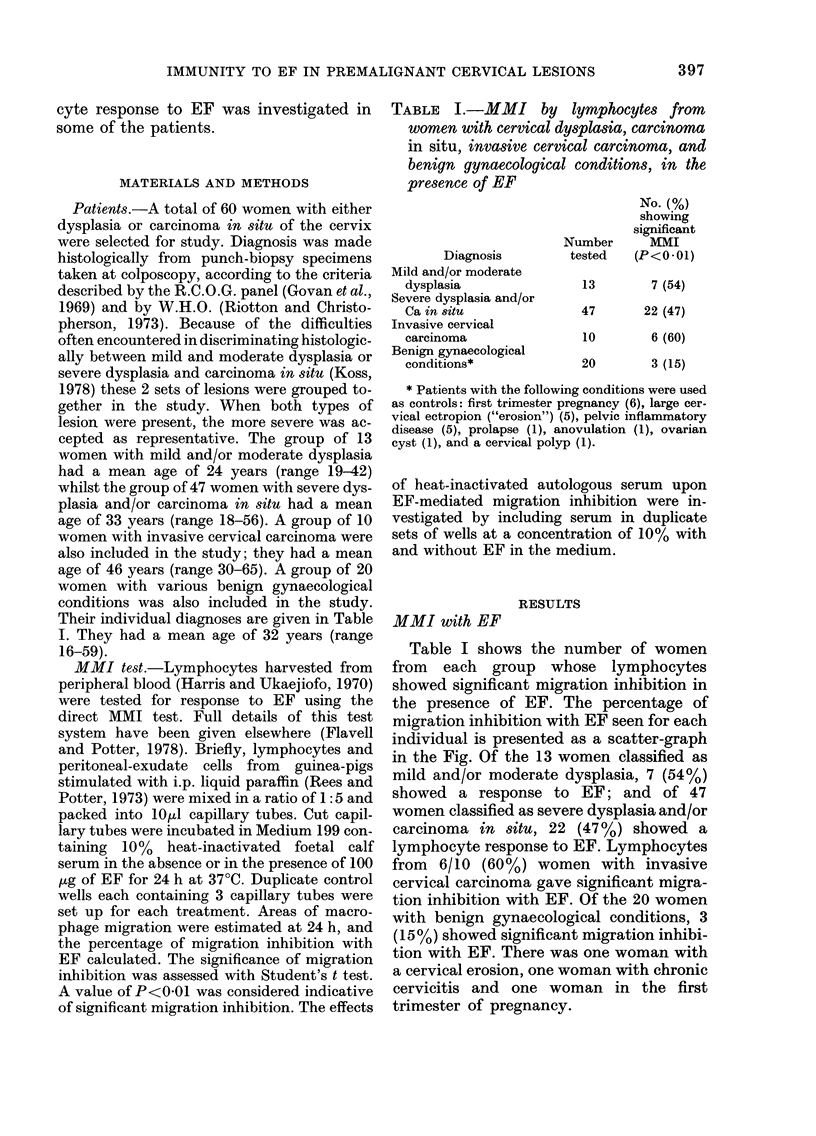

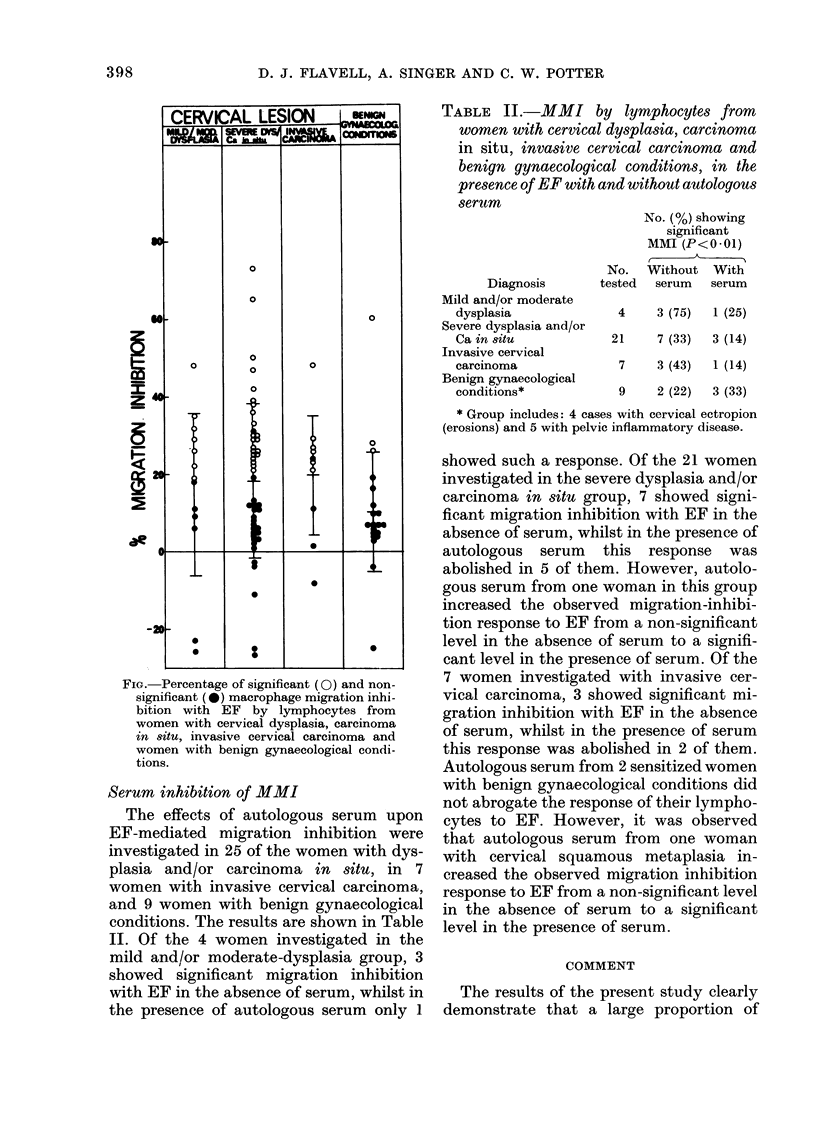

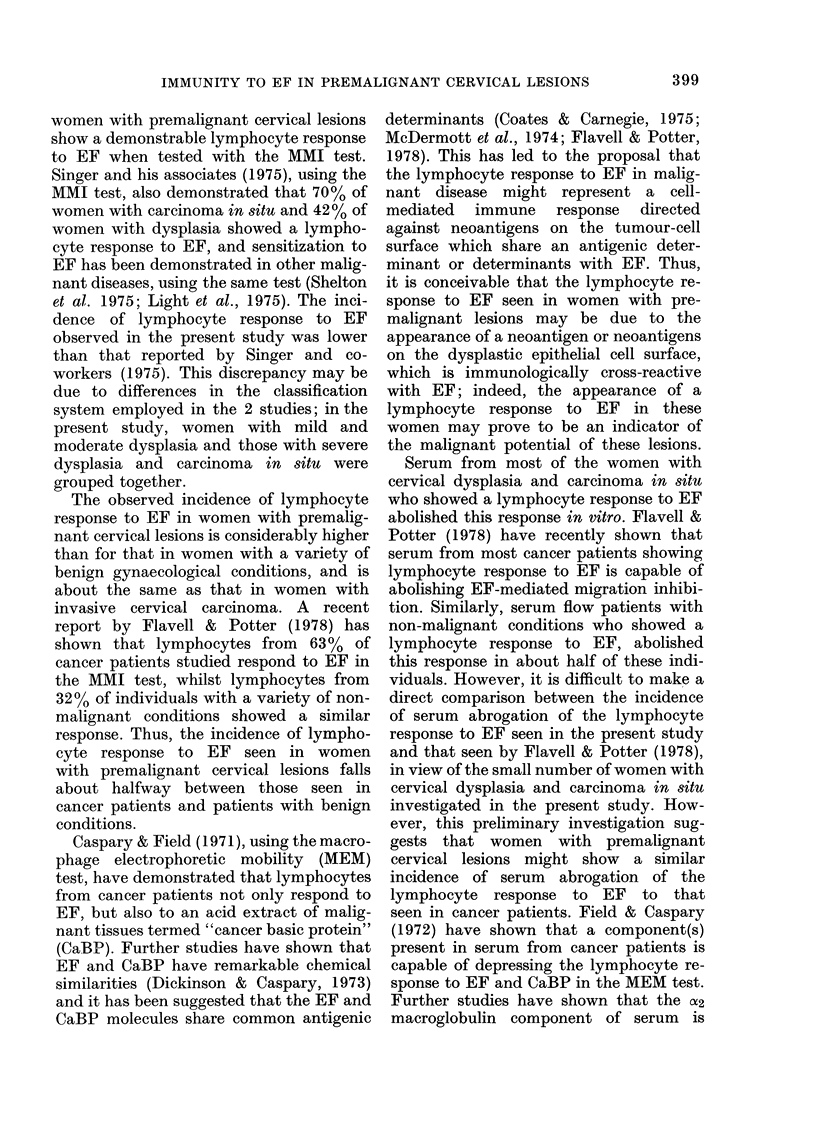

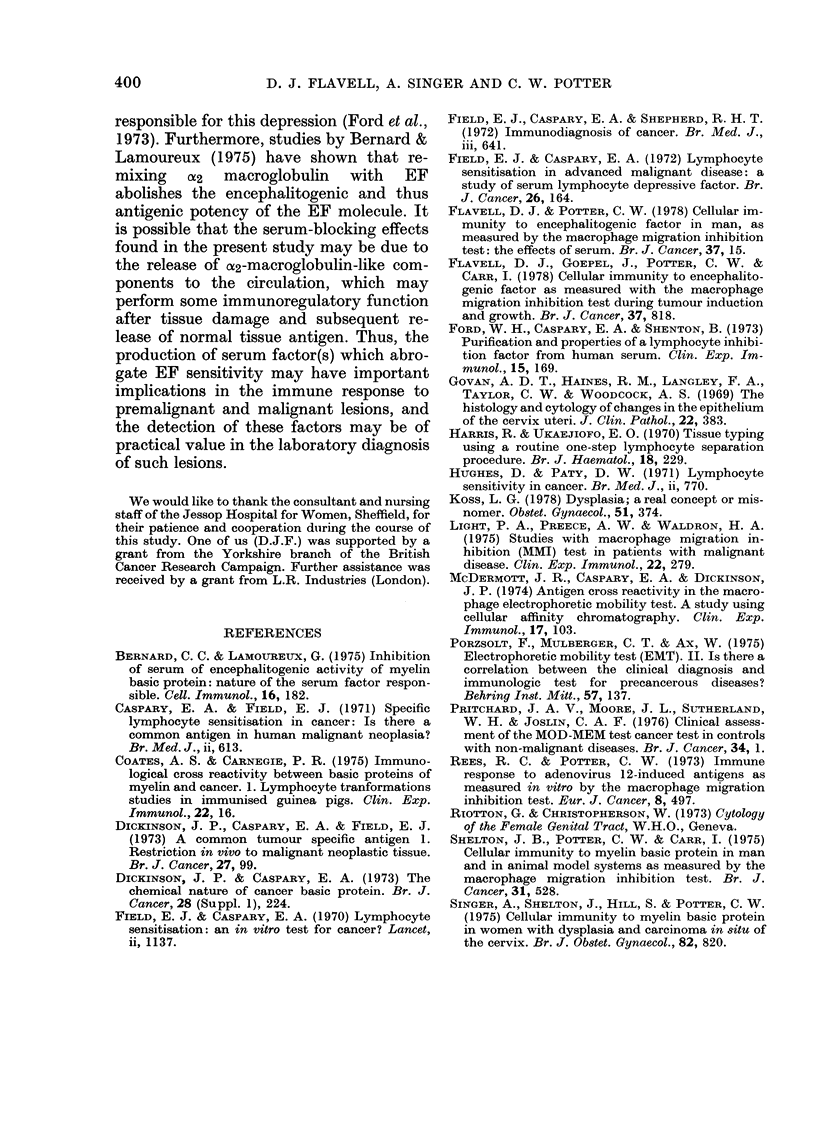


## References

[OCR_00472] Bernard C. C., Lamoureux G. (1975). Inhibition by serum of encephalitogenic activity of myelin basic protein: nature of the serum factor responsible.. Cell Immunol.

[OCR_00478] Caspary E. A., Field E. J. (1971). Specific lymphocyte sensitization in cancer: is there a common antigen in human malignant neoplasia?. Br Med J.

[OCR_00484] Coates A. S., Carnegie P. R. (1975). Immunological cross-reactivity between basic proteins of myelin and cancer. I. Lymphocyte transformation studies in immunized guinea-pigs.. Clin Exp Immunol.

[OCR_00491] Dickinson J. P., Caspary E. A., Field E. J. (1973). A common tumour specific antigen. I. Restriction in vivo to malignant neoplastic tissue.. Br J Cancer.

[OCR_00497] Dickinson J. P., Caspary E. A. (1973). The chemical nature of cancer basic protein.. Br J Cancer Suppl.

[OCR_00512] Field E. J., Caspary E. A. (1972). Lymphocyte sensitization in advanced malignant disease: a study of serum lymphocyte depressive factor.. Br J Cancer.

[OCR_00507] Field E. J., Caspary E. A., Shepherd R. T. (1972). Immunodiagnosis of cancer.. Br Med J.

[OCR_00524] Flavell D. J., Goepel J., Potter C. W., Carr I. (1978). Cellular immunity to encephalitogenic factor as measured by macrophage migration inhibition during tumour induction and growth.. Br J Cancer.

[OCR_00518] Flavell D. J., Potter C. W. (1978). Cellular immunity to encephalitogenic factor in man as measured by the macrophage migration inhibition test: the effects of serum.. Br J Cancer.

[OCR_00531] Ford W. H., Caspary E. A., Shenton B. (1973). Purification and properties of a lymphocyte inhibition factor from human serum.. Clin Exp Immunol.

[OCR_00537] Govan A. D., Haines R. M., Langley F. A., Taylor C. W., Woodcock A. S. (1969). The histology and cytology of changes in the epithelium of the cervix uteri.. J Clin Pathol.

[OCR_00543] Harris R., Ukaejiofo E. O. (1970). Tissue typing using a routine one-step lymphocyte separation procedure.. Br J Haematol.

[OCR_00548] Hughes D., Paty D. W. (1971). Lymphocyte sensitivity in cancer.. Br Med J.

[OCR_00552] Koss L. G. (1978). Dysplasia. A real concept or a misnomer?. Obstet Gynecol.

[OCR_00556] Light P. A., Preece A. W., Waldron H. A. (1975). Studies with the macrophage migration inhibition (MMI) test in patients with malignant disease.. Clin Exp Immunol.

[OCR_00562] McDermott J. R., Caspary E. A., Dickinson J. P. (1974). Antigen cross-reactivity in the macrophage electrophoretic mobility test. A study using cellular affinity chromatography.. Clin Exp Immunol.

[OCR_00581] Rees R. C., Potter C. W. (1973). Immune response to adenovirus 12-induced tumour antigens, as measured in vitro by the macrophage migration inhibition test.. Eur J Cancer.

[OCR_00591] Shelton J. B., Potter C. W., Carr I. (1975). Cellular immunity to myelin basic protein in man and in animal model systems as measured by the macrophage migration inhibition test.. Br J Cancer.

[OCR_00598] Singer A., Shelton J., Hill S., Potter C. (1975). Cellular immunity to human basic myelin protein in women with dysplasia and carcinoma in situ of the cervix.. Br J Obstet Gynaecol.

